# COGNITIVE TRAJECTORIES IN MIDDLE-AGED AMERICANS, 1995–2018

**DOI:** 10.1093/geroni/igad104.2784

**Published:** 2023-12-21

**Authors:** Rebecca Brown, Xi Zuo, Corey McMillan, Shana Stites, Irma Elo, Kenneth Langa, Jason Karlawish, Dawei Xie

**Affiliations:** University of Pennsylvania, Philadelphia, Pennsylvania, United States; University of Pennsylvania, Philadelphia, Pennsylvania, United States; University of Pennsylvania, Philadelphia, Pennsylvania, United States; University of Pennsylvania, Philadelphia, Pennsylvania, United States; University of Pennsylvania, Philadelphia, Pennsylvania, United States; University of Michigan, Ann Arbor, Michigan, United States; University of Pennsylvania, Philadelphia, Pennsylvania, United States; University of Pennsylvania, Philadelphia, Pennsylvania, United States

## Abstract

Given evidence that Alzheimer’s disease and related dementias (ADRD) develop over decades, middle age is increasingly recognized as a key life course period to target modifiable risk factors for cognitive decline and ADRD. Epidemiological trends show that the prevalence of chronic conditions that are risk factors for ADRD has increased in middle-aged adults over the past several decades, but it is unknown if cognitive trajectories have worsened in middle-aged people over this time period. We conducted a retrospective cohort study using data from 19,660 adults ages 50-64 enrolled from 1995-2018 in the longitudinal nationally representative Health and Retirement Study, followed through 2018. We fit group trajectory models to describe changes in cognition over time based on Telephone Interview for Cognitive Status scores and used mixed effects models to investigate change in slope adjusted for key risk factors (age, gender, race/ethnicity, education, net worth, chronic medical conditions, body mass index, age cohort). We identified four distinct groups of participants with differing start points in their cognitive trajectories. In adjusted analyses, trajectories of cognitive decline were steeper among racial/ethnic minorities, persons with lower education, lower net worth, history of stroke, and earlier age cohort. The effect of age cohort on the rate of decline was similar across racial/ethnic groups but differed across education and net worth groups. These findings suggest that cognitive trajectories among middle-aged adults have improved since the mid-1990s. However, disparities in cognitive trajectories persist among racial/ethnic minorities and those with lower education and net worth.

